# Preferences in the Willingness to Download a COVID-19 Contact Tracing App in the Netherlands and Turkey: Experimental Study

**DOI:** 10.2196/37891

**Published:** 2022-07-28

**Authors:** Frans Folkvord, Lutz Peschke, Yasemin Gümüş Ağca, Karlijn van Houten, Giacomo Stazi, Francisco Lupiáñez-Villanueva

**Affiliations:** 1 Tilburg School of Humanities and Digital Sciences Tilburg University Tilburg Netherlands; 2 PredictBy PredictBy Research Barcelona Spain; 3 Department of Communication and Design Bilkent University Ankara Turkey; 4 Department of Information and Communication Science Universitat Oberta de Catalunya Barcelona Spain

**Keywords:** COVID-19 tracing app, willingness to download, discrete choice task, pandemic, mitigation strategies, COVID-19, health application, mobile health, gaming, tracing application, digital health, data protection

## Abstract

**Background:**

Despite the worldwide growth in using COVID-19 contact tracing apps (CTAs) and the potential benefits for citizens, governments, health care professionals, businesses, and other organizations, only a few studies have examined the factors affecting the levels of willingness to download a CTA.

**Objective:**

This study aimed to investigate individuals’ preferences in the willingness to download a health app.

**Methods:**

We conducted an experimental study in 2 countries, the Netherlands (N=62) and Turkey (N=83), using 4 different vignettes (ie, data protection, manufacturer, reward, and gaming models) with different attributes. Participants were randomly assigned to 1 of the conditions within the vignettes.

**Results:**

The results showed that data protection and gaming elements are factors that influence the willingness to download a COVID-19 CTA. More specifically, we see that data protection is an important factor explaining the willingness to download the app in Turkey, whereas including gaming elements significantly affects the willingness to download the app in the Netherlands.

**Conclusions:**

COVID-19 CTAs are highly promising to reduce the spread of the virus and make it easier to open up society faster, especially because they can be used quickly and share information rapidly. COVID-19 CTA developers must ensure that their apps satisfactorily and sufficiently address ethical considerations, even in times of crisis. Furthermore, integrating gaming elements in the CTA could enhance the willingness to download the CTA.

## Introduction

### Background

The COVID-19 pandemic affected human health tremendously, as well as social and economic life, increasing the urge to find effective measures to start reviving public life as soon as possible while minimizing the risk of infection and hospitalization of patients [1]. Current developments across the world show that one strategy might be through technology, accompanied by mobile apps [2,3]. Considering that COVID-19 has proven to be highly contagious, especially newer variants such as Delta and Omicron [4]—sometimes without the carrier experiencing symptoms and infecting several others before actually testing positive—it is important to trace contacts and identify individuals who had close contact with the carrier.

Normally, this tracking is accomplished through a personal interview with the infected individual, establishing the people who have been in (close) contact with the carrier, and subsequently developing strategies to mitigate further spreading. However, it is difficult for people to accurately remember all the persons that they have been in close contact with, including those who cannot be identified because they are unknown to the carrier. Moreover, considering the rapidly increasing numbers of infected people in a majority of the countries worldwide at the moment, conducting the interviews manually would require a considerable workforce of trained individuals to hold the interviews effectively, which is a highly costly exercise and has shown to be ineffective in times of high infection rates [5].

Research has shown that COVID-19 contact tracings apps (CTAs) could mitigate the current pandemic by informing people instantly when they have been in close contact with an infected individual [5-9]. CTAs have been implemented in nearly all countries for the identification of pandemic hot spots, supporting collaborative information processes between the public and health authorities about critical contacts with infectious citizens. One of the main goals is to initiate knowledge circulation between the 5 systems of the Quintuple Helix [10], which includes the decision processes of policy makers. According to Oldeweme et al [6], the willingness to download processes of CTAs are fostered by the reduction of uncertainties—perceived privacy and performance risks—through trust in governments and public participation but do not reduce social risks and health-related COVID-19 concerns. Although these new possibilities promise several benefits for mitigating the pandemic, the actual willingness to download and the long-term use of mobile health (mHealth) apps is rather low and lags behind their potential [11]. As a result, despite the promising effects of the CTAs to mitigate the pandemic, there is insufficient evidence supporting the willingness to download preferences for these apps, although willingness to download is the first, and therefore important, step. This lack of evidence urges us to take a step back to better understand which factors influence the willingness to download CTAs [12,13].

A first step in this endeavor is to understand which factors affect people’s willingness to download CTAs. Considering the limited understanding of the general cognitive motivators that trigger people’s willingness to download health apps, especially in times of crisis, it is important to examine which attributes of health apps are preferred when downloading CTAs. An extensive systematic literature review [14] has shown that, in support of the Technology Acceptance Model, 8 key themes can be categorized for clinicians’ adoption of mHealth tools: usefulness, ease of use, design, compatibility, technical issues, content, personalization, and convenience. In addition, from a patient’s perspective [15], usefulness, ease of use, data-related factors, monetary factors, technical issues, and user experience were considered to be important factors for the adoption of an mHealth app.

Furthermore, intrinsic motivation to use such a health app is considered a strong predictor of actual willingness to download and use [16]. In addition, Salomoni et al [17] point out that rethinking how the users receive and interact with a health app is important to better understand the role of diegetic interfaces, which should also be taken into account when developing a CTA. Without the proper comprehension of the cognitive motivators that explain the willingness to download and use mHealth apps, it would be very difficult to establish the effectiveness of mHealth apps and fully understand individuals’ use of such apps.

### Attributes Explaining Preferences in the Willingness to Download CTAs

The rising use of mHealth apps threatens to change the way substantial amounts of health data will be managed, with a paradigm shift from mainframe systems located in the facilities of health care providers to apps on mobile phones and data stored in shared cloud services [18,19]. According to Klar and Lanzerath [20], besides the challenges of effectiveness, technological problems and the risks of privacy and equity have to be considered. Ryan [21] has, for example, evaluated South Korea’s digital tracing app through the lens of 4 human rights principles to determine if this response was ethically justifiable (ie, necessary, proportional, scientifically valid, and time-bounded) and concluded that the Korean digital CTA was scientifically valid and proportionate—meeting the necessity requirement—but it was too vaguely defined to meet the time-boundedness requirement. More specifically, the prerequisite of an ethical deployment of CTAs is voluntariness, starting with deciding to carry a smartphone, choosing to download and install a CTA, leaving the CTA operating in the background all the times, and finally, sharing contact logs when tested positive. Peschke et al [22] discussed the voluntariness of CTA deployment in different countries. Based on an analysis of more than 35 CTAs worldwide [23], 2 main categories of CTA deployment could be identified. First, for the participation of the public life, such as entering university campuses and shopping malls in Turkey, an individual Hayat Eve Sığar (Turkish for “life fits into home”) code has to be generated and presented to the gatekeepers. In these apps, the data of the users are collected in a central place and sometimes shared with other state institutions. If COVID-19 is detected within the passengers of the same vehicle or at the same location within the next 14 days, everyone at that place, family doctors, and the filiation team are informed [24]. Second, Google and Apple have developed a system called Exposure Notifications System to bring out anonymized identity information to the users’ environment via Bluetooth. The importance of data protection and privacy issues regarded by potential users of the CTAs have not been sufficiently understood yet.

A recent study has shown that privacy perceptions are related to the use of mHealth apps, in which people with more concerns about the secondary use of their personal data were less likely to use certain mHealth apps [25]. In addition, the manufacturer of a health app can be considered a heuristic in the consumers’ willingness to download a CTA. There is reason to assume that this factor may be an important, as the industry has struggled with its public image over the past few decades. Companies need to negotiate a tension between, on the one hand, striving for optimal health care and, on the other hand, striving for profit [26]. In the eyes of the public, it is not always clear that the industry has the patients’ interests at heart [27]. Therefore, people would probably prefer if an mHealth app is manufactured by the government than by a company.

By contrast, the Edelman Trust Barometer [28] reveals that business is the only institution that is understood to be competent and ethical by the public in 18 of the 27 countries evaluated. The public is more likely to trust in business as it acts as the guardian of information quality, embraces sustainable practices, and provides robust COVID-19 health and safety response [28]. However, the Netherlands noticed an increase of trust in both business and government [28]. The Edelman Trust Barometer did not capture data from Turkey, but the result of 2 studies conducted in 2020 show that the pandemic crisis revealed 2 different results. Bostan et al [29] conclude that the public trusts the authorities and the accuracy of the decisions taken by the state to combat the pandemic. However, Tanca et al [30] perceive the ambiguity and capriciousness of the public’s reactions as a result of the uncertainty of both the government and national economy. A special report about trust and coronavirus published by Edelman Trust Barometer [31] reveals that 85% of the respondent want to hear more from scientists and less from politicians.

Lastly, it is worth emphasizing the importance of the design of the CTA when participants are downloading apps. Web-based gaming studies have shown that elements and features with clear goals at every step [32], immediate feedback [33-35], and balance between challenge and skill [36] grounded in the flow theory [37] are considered key elements of the willingness to download apps.

Finally, since last year, the majority of the public has prioritized increasing their own media and information literacy as well as science literacy, but only 26% of the respondents have good information hygiene—considering news engagement, avoiding information echo chambers, verifying information, and not amplifying unvetted information [28].

### Hypotheses

In this study, we conducted 4 small experimental studies based on vignettes in 2 different countries (the Netherlands and Turkey). In the vignette study, we manipulated the manufacturer (government vs company), data protection (data protection vs no information), reward (no reward vs voucher as a reward), and gaming (no gaming elements vs gaming elements) variables and assessed the likelihood of downloading a COVID-19 CTA to test the following hypothesis:

1. Participants who are exposed to a COVID-19 CTA developed by a government will be more willing to download the app than participants who were exposed to a COVID-19 CTA that is developed by a company (Hypothesis [H]1).
2. Participants who are exposed to a COVID-19 CTA whereby there was communication that the data is protected by law will be more willing to download the app than participants who are exposed to a COVID-19 CTA whereby no information was given about the data protection (H2).
3. Participants who are exposed to a COVID-19 CTA and will receive a reward based on scientific evidence will be more willing to download the app than participants who are exposed to a COVID-19 CTA and will receive no rewards (H3).
4. Participants who are exposed to a COVID-19 CTA that includes gaming elements will be more willing to download the app than participants who are exposed to a COVID-19 CTA without any gaming elements (H4).

## Methods

### Design

In this study, we conducted 4 small experimental studies through a web-based questionnaire using vignettes to simulate real-life situations. The use of a web-based questionnaire provided completion time data to support the internal validity checks and enabled an accurate record of the time taken to complete the surveys. There were 2 rounds of cognitive testing (N=12) undertaken in the Netherlands to check the participants’ comprehension of information when making choices. These pretests confirmed that a study based on the questionnaire was acceptable and understandable for participants, after some minor revisions in the explanation of the task.

### Procedure

All survey participants were informed about the overall study goals and procedures. First, participants were asked to provide sociodemographic information, including their age group, gender, education, and employment status. Subsequently, an introduction to the vignette studies provided an explanation of what was expected from the participants. One example of the vignette is the following, whereby the last sentence is the part of the script that varied among the conditions.

Imagine that an app is presented to you to mitigate the pandemic, by providing you detailed information about the people that you have been around with, how close, and for how long, that are infected by the COVID virus, or not. Based on the collected data, the app will provide tailored advice to improve your health. We will present you some pairs of options and will ask you to select the one that you would prefer to use the app or not. Importantly, the app is developed by the government.

Subsequently, participants were asked to score the likelihood that they would download the app and whether they would download the app. The other vignettes entailed exactly the same text, except the last sentence was replaced by the specific condition the participant was randomly allocated to (government vs company, data protection vs no information, no reward vs voucher as a reward, or no gaming elements vs gaming elements).

### Ethics Approval

Only those who agreed to participate in the study gained access to the web-based survey. The approval of the Ethical Committee of the Tilburg School of Humanities and Digital Sciences to conduct the experiment was obtained (REDC 2021.73). Through signing informed consent, participants were ensured that their data would remain confidential, and they were told that they could cease participation at any moment. All participants participated anonymously, and the collected data was stored in a dark archive at the Tilburg University.

### Participants

For this study, we used data collected from 2 different countries (Turkey and the Netherlands). This study was part of a larger project, whereby multiple experiments were conducted. After the participants finished the discrete choice experiment, they also participated in a separate experiment that is reported in a separate paper. The data in the Netherlands (N=62) and Turkey (N=83) were collected through a web-based survey administered through Qualtrics (SAP America Inc). Participant characteristics are shown in Table 1.

**Table 1:** Descriptive information about the participants per country.

Characteristic			Netherlands (N=62)		Turkey (N=83)
Gender, women, n (%)			31 (50)		43 (52)
Age (year), mean (SD)			23.92 (7.45)		21.87 (2.46)
**Educational level, n (%)**					
	Primary education	3 (5)		0 (0)	
	High school diploma	3 (5)		10 (12)	
	Some years of university	28 (45)		61 (73)	
	University degree	18 (29)		10 (12)	
	Post-graduate degree	10 (16)		2 (2)	
**Employment status, n (%)**					
	Employed/self-employed	11 (18)		12 (14)	
	Unemployed	7 (11)		0 (0)	
	Student	44 (71)		71 (86)	
	Retired	0 (0)		0 (0)	
	Not working due to illness or disability	0 (0)		0 (0)	
	Another reason for not being in the labor force	0 (0)		0 (0)	
**Health app use, n (%)**					
	No use	27 (44)		30 (36)	
	1 time	8 (13)		18 (22)	
	2 times	11 (18)		18 (22)	
	3 times	7 (11)		6 (7)	
	4 times	3 (5)		4 (5)	
	5 times	0 (0)		3 (4)	
	>5 times	6 (10)		4 (5)	
Health consciousness, mean (SD)			3.77 (0.68)		3.93 (0.83)
Health information orientation, mean (SD)			2.99 (0.82)		3.48 (0.92)
eHealth literacy, mean (SD)			2.85 (0.96)		3.09 (1.00)

### Measures

#### Dependent Variables

Willingness to download the app was measured through the question “Please indicate on a scale of 1 to 10 how likely it is that you would download the app. Please place yourself at a point on this scale where ‘0’ indicates that you would ‘definitely not download the app,’ ‘10’ indicates that you would ‘definitely download the app,’ and the remaining numbers indicate something in between these 2 positions.” whereby participants could answer on a visual analog scale.

Intention to download the app was measured through the question “Would you download the app?” whereby participants could answer yes or no.

#### Intrapersonal Factors

Health app use was measured by asking how often the participant used a health app, varying from 0 (never) to 6 (more than 5 times), and how much time the participant spent using a health app in the last week, varying from 0 (0 hours) to 6 (more than 1 hour).

Health consciousness was measured using 5 statements that were rated on a 5-point scale (from 1 representing strongly disagree to 5 representing strongly agree) [38]. The reliability of the scale was high (*α*=.82).

Health information orientation was measured using 8 statements rated on a 5-point scale (from 1 representing strongly disagree to 5 representing strongly agree) [38]. The reliability of the scale was high (*α*=.87)

eHealth literacy was measured using 8 statements rated on a 5-point scale (from 1 representing strongly disagree to 5 representing strongly agree) [39]. The reliability of the scale was high (*α*=.95).

### Statistical Analyses

Several analyses of covariance (ANCOVAs) were conducted to assess the participants’ likelihood to download the app—first, for all participants and subsequently, for each country. Next, we conducted logistic regression analyses to assess the participants’ probability to download the app.

## Results

### Developer Models

The first ANCOVA tested if participants who were exposed to a COVID-19 CTA developed by a government were more willing to download the app than participants who were exposed to a COVID-19 CTA developed by a company, whereby age, gender, education, health consciousness, health information orientation, and eHealth literacy were included as covariates in the analysis. No effects were found for the developer (*F*_7,137_=1.307; *P*=.26). For age (*F*_1,137_=9.848; *P*=.002) and eHealth literacy (*F*_1,137_=7.047; *P*=.009), we found significant relationships. For the other factors, we found no significant differences.

Next, the logistic regression analysis showed that there was no effect of the developer on whether the participants would download the app (*P*=.32). For age (*P*<.001) and education (*P*=.02), we found significant relations to whether the participants would download the app.

A separate ANCOVA for the Netherlands showed that no effects were found for the developer (*F*_7,54_=1.638; *P*=.21). We found a significant relationship for age (*P*=.01). For the other factors, we found no significant differences. In addition, the logistic regression analysis showed that there was no effect of the developer on whether the participants would download the app (*P*=.46). For age (*P=.*01) and education (*P*=.008), we found significant relations to whether the participants would download the app.

In Turkey, an ANCOVA showed that no effects were found for the developer as well (*F*_7,75_=0.094; *P*=.76). For the other factors, we found no significant differences. Furthermore, the logistic regression analysis showed that there was no effect of the developer on whether the participants would download the app (*P*=.49). For age (*P=.*02) and eHealth literacy (*P*=.01), we found significant relations to whether the participants would download the app.

### Data Protection Models

The second ANCOVA tested if participants who were exposed to a COVID-19 CTA developed whereby data was protected by European legislation were more willing to download the app than participants who were exposed to a COVID-19 CTA whereby no information was given about the data protection, whereby age, gender, education, health consciousness, health information orientation, and eHealth literacy were included as covariates in the analysis. The results showed that participants who were exposed to a COVID-19 CTA developed whereby data was protected by law (mean 6.93, SD 2.44) were significantly more willing to download the app than participants who were exposed to a COVID-19 CTA whereby no information was given about the data protection (mean 6.93, SD 2.44; *F*_7,137_=19.125; *P*>.001). For the other factors, we found no significant differences. The logistic regression analysis showed that there was a significant effect of data protection on whether the participants would download the app (*P*<.001). The probability that a participant would download the app would be 3.886 times greater when the data was protected by EU legislation than when no information was provided about what would be done with the data. For the other factors, we found no significant relations.

Separate analyses for the Netherlands showed that participants who were exposed to a COVID-19 CTA developed whereby data was protected by law were not more likely to download the app than participants who were exposed to a COVID-19 CTA whereby no information was given about the data protection (*F*_7,54_=1.130; *P*=.29). For the other factors, we found no significant differences. The logistic regression analysis showed that there was a significant effect of data protection on whether the participants would download the app (*P*=.03). The probability that a participant would download the app would be 3.327 times greater when the data was protected by law than when no information was provided about what would be done with the data. For the other factors, we found no significant relations.

In Turkey, participants who were exposed to a COVID-19 CTA developed whereby data was protected by law were significantly more willing to download the app (mean 7.27, SD 2.52) than participants who were exposed to a COVID-19 CTA whereby no information was given about the data protection (mean 4.17, SD 3.09; *F*_7,75_=24.584; *P*>.001). For the other factors, we found no significant differences. The logistic regression analysis showed that there was a significant effect of data protection on whether the participants would download the app (*P*=.007). The probability that a participant would download the app would be 3.847 times greater when the data was protected by law than when no information was provided about what would be done with the data. For the other factors, we found no significant relations.

### Reward Models

The next ANCOVA tested if participants who were exposed to a COVID-19 CTA and would receive a reward if they adopted the app were more willing to download the app than participants who were exposed to a COVID-19 CTA and would receive no rewards, whereby age, gender, education, health consciousness, health information orientation, and eHealth literacy were included as covariates in the analysis. The results showed that participants who were exposed to a COVID-19 CTA and would receive a reward if they adopted the app were not more willing to download the app than participants who were exposed to a COVID-19 CTA and would receive no rewards (*F*_7,137_=0.324; *P=.*57). For the other factors, we found no significant differences. In addition, the logistic regression analysis showed that there was no effect of reward on whether the participants would download the app (*P*=.93). For age (*P=.*002), health consciousness (*P*=.04), health information orientation (*P*<.001), and eHealth literacy (*P*=.003), we found significant relations to whether the participants would download the app.

Separate analyses for the Netherlands showed that participants who were exposed to a COVID-19 CTA and would receive a reward if they adopted the app were not more willing to download the app than participants who were exposed to a COVID-19 CTA and would receive no rewards (*F*_7,54_=0.322; *P=.*57). For the other factors, we found no significant differences. The logistic regression analysis showed that there was no effect of reward on whether the participants would download the app (*P*=.24). For gender (*P*=.05), age (*P*=.003), health consciousness (*P*=.006), health information orientation (*P*=.004), and eHealth literacy (*P*=.003), we found significant relationships to whether the participants would download the app. In Turkey, we found similar results for the reward (*P=.*74) and no other significant relations.

### Gaming Models

The next ANCOVA tested if participants who were exposed to a COVID-19 CTA that included gaming elements were more willing to download the app than participants who were exposed to a COVID-19 CTA without any gaming elements, whereby age, gender, education, health consciousness, health information orientation, and eHealth literacy were included as covariates in the analysis. The results showed that participants who were exposed to a COVID-19 CTA where gaming elements were included (mean 6.46, SD 2.28) were significantly more willing to download the app than participants who were exposed to a COVID-19 CTA without any gaming elements (mean 5.46, SD 2.73; *F*_7,137_=6.603; *P*=.01). We found a significant relationship for age (*F*_1,137_=6.008; *P*=.02). For the other factors, we found no significant differences. Next, the logistic regression analysis showed that there was no effect of gaming on whether the participants would download the app (*P*=.15). For education (*P=.*03) and health information orientation (*P*=.02), we found significant relations to whether the participants would download the app.

Separate analyses for the Netherlands showed that participants who were exposed to a COVID-19 CTA where gaming elements were included (mean 6.51, SD 2.07) were significantly more willing to download the app than participants who were exposed to a COVID-19 CTA without any gaming elements (mean 4.87, SD 2.27; *F*_7,54_=26.755; *P*<.001). Furthermore, we found a significant relationship for age (*F*_1,54_=13.399; *P*<.001). For the other factors, we found no significant differences. Next, the logistic regression analysis showed that there was a significant effect of gaming on whether the participant would download the app (*P*=.006). Participants who were exposed to a COVID-19 CTA with gaming elements were 20.516 times more likely to download the app than participants who were exposed to a COVID-19 CTA without gaming elements. For age (*P*=.03), education (*P=.*02), health consciousness (*P*=.04), and eHealth literacy (*P*=.03), we found significant relations to whether the participants would download the app. In Turkey, we found no significant differences between the gaming conditions (*F*_7,75_=2.380; *P*=.57) and for the other factors. The logistic regression analysis showed that there was no effect of gaming on whether the participants would download the app (*P*=.62). No other factors were significantly related to whether the participants would download the app.

## Discussion

### Principal Findings

The results showed that data protection and gaming elements are important factors that affect the willingness to download a COVID-19 CTA, thereby supporting H2 and H4. For the developer and reward, we found no significant differences between the participants, thus rejecting H1 and H3. Governments might not have a second chance to get an intervention right, especially because in times of crises, trust in politicians is an important factor to mitigate the crisis at stake. Governments, developers, and deployers must ensure that COVID-19 CTAs satisfactorily address the ethical questions that were set out [3]. According to the Organisation for Economic Cooperation and Development [40], citizens expect integrity, openness, and fairness in communication and knowledge transfer, where responsiveness and reliability are crucial factors. As a result, a positive perception of comprehension leads to an increase in acceptance and cooperative engagement.

### Comparison With Prior Work

Despite the worldwide growth in using COVID-19 CTAs and the potential benefits for all the actors within the Quintuple Helix to mitigate the pandemic more effectively and make us able to open up society sooner, only a few studies have examined the factors affecting the levels of willingness to download the apps. In this study, we investigated individuals’ preferences in the willingness to download a health app. Digital contact tracing via smartphone apps was established as a new public health intervention in many countries in 2020. Most of these apps are now at a stage where they need to be evaluated as public health tools, especially because this could provide us with important lessons for future events such as the current pandemic [3,41].

### Limitations

One of the limitations of the study is that we used a convenience sampling, whereby most of the participants were students from only 2 countries, thus reducing the generalizability of the outcomes. Considering that this study is part of a larger study where we will develop an actual CTA that will be tested among a larger and more representative study in 5 different countries (Spain, the Netherlands, Finland, Germany, and Turkey), the generalizability will improve. Finally, gamification elements might have been judged differently if they were actually displayed or could be tried out in the experiment, increasing its effectiveness, which unfortunately was not possible. In the next phase of the study, we will test gaming elements in more detail.

### Conclusions

Simply rolling out a CTA without ethical considerations is not acceptable and does not support the knowledge circulation in the Quintuple Helix. Even in a crisis, a “try everything” approach is dangerous when it ignores the real costs, including serious and long-lasting harms to fundamental rights and freedoms and the opportunity costs of not devoting resources to something else. As this pilot study shows, reassuring people that data protection regulations are put in place is an essential element for increasing the levels of willingness to download CTAs.

During the last few decades, mHealth initiatives have emerged at an accelerating pace; some have seen widespread willingness to download, whereas others have failed to provide sustained value [42]. These failings can be attributed to their design and implementation efforts that were initiated without a good understanding of the interdependencies between technology, societal and cultural values, and user experience in a health care setting, which has become highly apparent during the current pandemic and health care crisis. Many conceptual frameworks based on implementation science have been developed to evaluate and orient mHealth delivery. These frameworks highlight key factors that predict successful and sustainable mHealth technologies, although there is still limited understanding of the factors influencing the willingness to download these technologies. The urgency of the ongoing public health crisis stimulated the rapid development of CTAs and other mHealth innovations, and this generated a substantial number of related publications. Their coverage of the essential design and implementation characteristics for eHealth innovation remains under investigated and needs to be further researched in future studies [41].

## Abbreviations

ANCOVA: analysis of covariance
CTA: contact tracing app
H: hypothesis
mHealth: mobile health
